# High frequency repetitive transcranial magnetic stimulation for auditory verbal hallucinations in schizophrenia-spectrum disorders: a naturalistic study

**DOI:** 10.3389/fpsyt.2025.1551901

**Published:** 2025-06-05

**Authors:** Virginie Moulier, Charlotte Lemonnier, Sonia Dollfus, Olivier Guillin, Maud Rothärmel

**Affiliations:** ^1^ Centre Hospitalier du Rouvray, Service Hospitalo-Universitaire de Psychiatrie, Centre d’Excellence Thérapeutique, Sotteville-lès-Rouen, France; ^2^ Etablissement Public de Santé (EPS) Ville Evrard, Centre de Recherche Clinique, Neuilly-sur-Marne, France; ^3^ Inserm UMR-S 1237 PhIND, Presage team, GIP Cyceron, Caen, France; ^4^ Normandie Univ, Université de Caen Normandie (UNICAEN), UFR de Médecine, Caen, France; ^5^ CHU de Caen, Service de Psychiatrie, Caen, France; ^6^ Fédération Hospitalo-Universitaire (FHU) A2M2P- CHU, Caen, France; ^7^ Department of Psychiatry, Rouen University Hospital, Rouen, France; ^8^ Faculté de Médecine, Normandie University, Rouen, France; ^9^ INSERM U 1245, University of Rouen, Rouen, France

**Keywords:** schizophrenia, hallucinations, repetitive transcranial magnetic stimulation (rTMS), high frequency rTMS, left temporo-parietal junction region

## Abstract

**Background:**

This study investigates the efficacy of high frequency repetitive transcranial magnetic stimulation (rTMS) for auditory verbal hallucinations in patients with schizophrenia spectrum disorders in routine clinical practice.

**Methods:**

In this monocentric study, data were collected on patients with schizophrenia treated by rTMS for resistant auditory verbal hallucinations from May 2020 to May 2024. Treatment efficacy was regularly assessed.

**Results:**

The data of 65 patients were collected. There was a significant improvement in the Auditory Hallucination Rating Scale (AHRS, *p*<0.001), in the Brief psychiatric Rating scale (BPRS, *p*<0.001) and in the Clinical Global Impression-Improvement *(p*<0.001) scores over time (from baseline up to six months). The maximum response rate (40%) was obtained after nine weeks of rTMS (on average (SD), after 30.6 (7.8) rTMS sessions). The responders were significantly younger than non-responders (p=0.002). The good tolerance of the rTMS treatment allowed excellent compliance: only seven patients (10.8%) asked to stop rTMS or were non-compliant.

**Conclusion:**

These data show the clinical interest and the good tolerance of rTMS in daily practice in patients with schizophrenia suffering from auditory verbal hallucinations.

## 1 Introduction

Characterized by the perception of voices without external stimuli (1), the auditory verbal hallucinations (AVH) are a key symptom of schizophrenia, concerning 50 to 70% of patients (2, 3). Although first-line antipsychotic drugs can help alleviate AVH, in 20-40% of the cases, the response to treatment is insufficient (4, 5). AVHs are considered as treatment-resistant when symptoms are present in patients following a failure to respond to at least two appropriate pharmacological treatments (6). In case of treatment-resistant AVHs, medication switch is recommended especially for clozapine, which is considered the most efficient antipsychotic agent in resistant patients. However 40-70% of treatment-resistant patents achieve only poor or partial response to clozapine (7). In this case, the development of alternative approaches are needed, such as noninvasive brain stimulation methods.

The use of neurostimulation techniques mostly lies on neuroimaging evidence of abnormal brain activity and connectivity underlying schizophrenia symptoms. Neuroimaging studies demonstrated that experiencing AVHs is associated with hyperactivity in frontal, temporal and parietal areas involved in speech generation and speech perception (8). Disrupted white matter integrity was found in the left arcuate fasciculus, indicating a decreased connectivity of the left fronto-temporal network in AVHs (9). In addition, a reduced grey matter volume in a large cluster in the left superior temporal gyrus was associated with greater AVHs severity (10). Consequently, the temporo-parietal junction has emerged as a natural target for neurostimulation studies.

Among these brain stimulation techniques, repetitive transcranial magnetic stimulation (rTMS) uses electromagnetic pulses to induce an electrical current in the underlying cortical tissue. Repetitive application of this stimulation modifies cortical activity (inhibition or excitation), generating modulation effects within the target region and its associated network. According to guidelines (11), low frequency rTMS of the left temporoparietal cortex (level C) may demonstrate possible efficacy in alleviating AVHs, with a greater efficacy in young patients and in females (12).

However, two recent meta-analyses questioned this efficacy. Including 11 randomized controlled trials (RCT) with rigorous inclusion criteria, a meta-analysis showed that 1-Hz rTMS targeting the left temporoparietal cortex had a moderate effect size, but they declared themselves unable to definitively support or refute the routine use of 1-Hz rTMS in treating AVH in clinical practice (13). Including 27 RCTs, another meta-analysis did not find a significant effect of rTMS on AVH, even in analyses including only low-frequency rTMS (14).

For several years, an alternative to the use of low frequencies has emerged to treat AVH. Indeed single rTMS sessions over the temporal lobe at low or high frequency have the same effect on cortical auditory event-related potentials (P50): both stimulation frequencies induced a decrease in P50 amplitude, considered as a marker of temporal cortex excitability (15). So, high-frequency rTMS applied to the temporal cortex could exert a neuromodulation effect comparable to that of low-frequency rTMS. In an open-label pilot study, 11 patients with schizophrenia were treated by two days of 20-Hz rTMS applied on the posterior part of the left superior temporal sulcus (16). Severity and frequency of AVH were significantly reduced. Two patients did not experience any AVH at six-month follow-up. This encouraging result was confirmed by a RCT, in which 59 patients were treated with active/sham 20-Hz rTMS applied over the left temporal cortex (17). Four 13-min rTMS sessions were performed with two sessions a day. The percentages of patients showing a decrease of more than 30% of Auditory Hallucination Rating Scale (AHRS) score significantly differed between the active (34.6%) and sham groups (9.1%) two weeks after the last rTMS session. However this difference was no longer significant in the longer term (three weeks and more). These previous studies showed promising results of high-frequency rTMS which had a double advantage: shorter session duration (13 versus 20 minutes) administered over a shorter period (two days versus two weeks). Indeed, about the same number of 20 Hz rTMS stimuli (10400) was administered to that routinely given at low frequency over two weeks.

However these studies raised several questions, such as: i) could the percentage of responders increase if the rTMS course lasted more than a week, especially for more severe patients?; ii) is-it possible to maintain the effect of rTMS with additional sessions following the initial phase (maintenance rTMS sessions)? Another relevant question is whether this effect of rTMS on AVH is also observed in clinical practice. Indeed, patients included in RCTs are rarely representative of real-world patient populations due to the application of strict selection criteria (18). In addition, in the RCT which demonstrated a transient effect of 20 Hz rTMS in schizophrenia, the rTMS target was determined by neuronavigation through magnetic resonance imaging (MRI) (17). However it is not always possible to perform an MRI, especially if the patient suffers from treatment-resistant schizophrenia and is agitated.

In this retrospective study, we therefore carried out a naturalistic study of rTMS as a treatment for AVH, with the aim of: (i) assessing changes in the severity of symptoms after rTMS over the 25 weeks of follow-up, as well as response rates, and, (ii) exploring potential moderators of treatment response in the naturalistic use of rTMS, by comparing sociodemographic and clinical characteristics between responders and non-responders.

## 2 Methods

### 2.1 Participants

In this naturalistic, open-label, retrospective and monocentric study, data was collected from clinical files of Rouvray Hospital in Sotteville-Lès-Rouen (France). Inclusion criteria were: adult patients with schizophrenia spectrum disorder (male or female) and presence of severe treatment-resistant AVHs despite treated with antipsychotic medication at efficient dose *(*≥600 mg chlorpromazine equivalent) and duration according to guidelines and despite the failure at least two previous medications with molecules from different pharmacological classes and who were treated with rTMS from May 2020 to May 2024. Consistent with the methodology of Dollfus et al. (17)’s study, patients were referred for rTMS, when their severity score of hallucinations on the Auditory Hallucination Rating Scale (AHRS) score exceeded 10.

Exclusion criteria were an intracranial or intracochlear metallic implant, pregnancy, and/or non-stabilized epilepsy. Patients with psychiatric and/or medical comorbidities were not excluded. Informed consent was obtained from all patients. They did not object to their data being used anonymously for research purposes. This study was conducted in accordance with the declaration of Helsinki and under French ethical law (public health code) that authorizes retrospective studies based on the exploitation of routine care data.

### 2.2 rTMS treatment

A MagPro R30 (MagVenture distributed by Mag2Health, Farum, Denmark) was used with a figure-8 coil to modulate the left temporo-parietal region, using the T3P3 site according to the International 10–20 system of electroencephalography (EEG) electrode positioning. As in the study of Dollfus et al. (17), the high-frequency protocol (20 Hz) consisted of 13 trains with a duration of 10 s and 200 pulses in each train. The stimulation intensity was at 80% of the resting Motor Threshold (rMT). The intertrain interval was 50 s, resulting in 2600 total pulses and a total duration of 13 min. Four 13-min rTMS sessions were performed, with 2 sessions a day. All patients started with an initial phase of four 13-min rTMS sessions per week (2 sessions a day with an hour interval; 2 days a week). Depending on the clinical severity of patients at baseline (assessed with the Clinical Global Impression (CGI)), this initial phase lasted between one and nine weeks, then was followed by a maintenance phase. During this maintenance phase, the number of rTMS sessions and their frequency were based on the patient’s clinical response, but also the patient’s wish. During this phase, the decrease in rTMS sessions frequency was gradual: with two sessions a week during four to eight weeks, then two sessions every two weeks during one to two months. If the patient’s clinical condition deteriorated during the maintenance phase, the spacing of rTMS sessions stopped and the frequency of four sessions per week resumed.

### 2.3 Clinical assessment

Treatment efficacy was regularly evaluated (baseline, after three, five, nine, 17 and 25 weeks of rTMS). Different scales routinely used in clinical practice were administered to the patients before and after initiating treatment: (i) the Auditory Hallucination Rating Scale (AHRS) to assess HAV (19); (ii) the Brief Psychiatric Rating Scale (18-item) to explore general psychopathology (20); (iii) the Clinical Global Impression (CGI) to study the patient’s global functioning (21). Side effects were also recorded. Response was defined as a decrease of at least 30% in the AHRS baseline score after the rTMS treatment (AHRS*_Baseline_*-AHRS*_After xx rTMS sessions_*)/AHRS*_Baseline_* * 100). This cutoff of 30% reduction in the AHRS was chosen because even minimal improvement can be clinically relevant for patients with treatment-resistant schizophrenia.

### 2.4 Statistical analyses

Statistical analyses were conducted using SPSS, version 29 (IBM, Armonk, NY, USA). Variables were reported as mean (standard deviation, SD) and range if quantitative, and as percentage if qualitative.

Anova with repeated measures was used to test whether AHRS and BPRS scores changed significantly over time. For exploratory purposes, patients were divided into two subgroups according to their clinical response after rTMS. Due to small subgroups size, non-parametric Mann-Whitney test was used to compare subgroups for quantitative outcomes. For categorical outcomes, comparisons between both subgroups relied on χ^2^ test or Fisher’ exact test as appropriate.

## 3 Results

### 3.1 Sociodemographic and clinical characteristics of the sample

The data of 65 patients with treatment-resistant AVH were collected. Sociodemographic and clinical characteristics of the total sample at baseline are reported in **Table 1**. Regarding their diagnosis, they suffered from: schizophrenia (86.2%) or schizoaffective disorder (13.8%). Except one patient, all were resistant to clozapine: they exhibited persistent AVHs despite clozapine for at least 6 weeks prior, with a plasma concentration ≥ 350 ng/ml. 49.21% of the patients were outpatients, and 50.8% were inpatients: 31.25% of inpatients were in high security unit, because they were considered dangerous for themselves or for others. 44.6% of the sample had a history of suicidal attempts. Regarding somatic comorbidities, 3.1% of the patients suffered from epilepsy. In addition, 4.6% had alcohol addiction and 6.2% had cannabis addiction (this low frequency is probably explained by the fact that half of the patients were hospitalized and unable to obtain alcohol and toxic substances). Regarding pharmacological treatment during rTMS course, the patients were taking either clozapine (75%), second-generation antipsychotics (15%) or a combination of first- and second-generation antipsychotics (10%).

**Table 1:** Characteristics of the sample and comparison between responders and non-responders after nine weeks of rTMS.

Variables	Total sample	Responders after nine weeks of rTMS	Non-responders after nine weeks of rTMS (+ dropout)	P value
**Sample size**	65	26	39	
**% of males**	70.8%	69.2%	73.1%	0.738
**Age**				**0.002**
mean (SD)	37.5 (13.3)	31.7 (12.8)	41.3 (12.4)	
median [range]	37.0 [17.0; 67.0]	29.0 [17.0; 67.0]	44.0 [20.0; 65.0]	
**Laterality : % right handedness**	90.3%	95.7%	87.2%	0.398 (Fisher exact)
**Number of rTMS sessions administered in nine weeks**				0.388
mean (SD)	30.6 (7.8)	29.6 (8.1)	32.0 (7.4)	
median [range]	36.0 [16.0; 36.0]	36.0 [16.0; 36.0]	36.0 [16.0; 36.0]	
**Baseline CGI severity**				0.645
mean (SD)	5.2 (1.0)	5.1 (1.1)	5.2 (1.0)	
median [range]	5.0 [4.0; 7.0]	5.0 [4.0; 7.0]	5.0 [4.0; 7.0]	
**Baseline AHRS score**				0.382
mean (SD)	29.4 (5.3)	30.0 (5.1)	28.9 (5.4)	
median [range]	30.0 [15.0; 39.0]	31.0 [19.0; 39.0]	30.0 [15.0;38.0]	
**Baseline BPRS score**				0.433
mean (SD)	43.6 (14.3)	45.5 (13.4)	42.4 (14.8)	
median [range]	43.0 [12.0; 84.0]	44.0 [25.0; 84.0]	43.0 [12.0; 72.0]	
**Nicotine addiction**	69.2%	73.1%	66.7%	0.583
**Pharmacological treatment**				
**Clozapine**	75.4%	84.6%	69.2%	0.158
**Antiepileptic drug**	46.2%	34.6%	53.8%	0.128
**Benzodiazepines**	42.2%	46.2%	39.5%	0.595

p-values correspond to the comparison between the two groups with Mann-Whitney tests. AHRS, Auditory Hallucination Rating Scale; BPRS, Brief Psychiatric Rating Scale; CGI, Clinical Global Impression. Bold value indicates significant.

### 3.2 Clinical evolution

**Figure 1:**
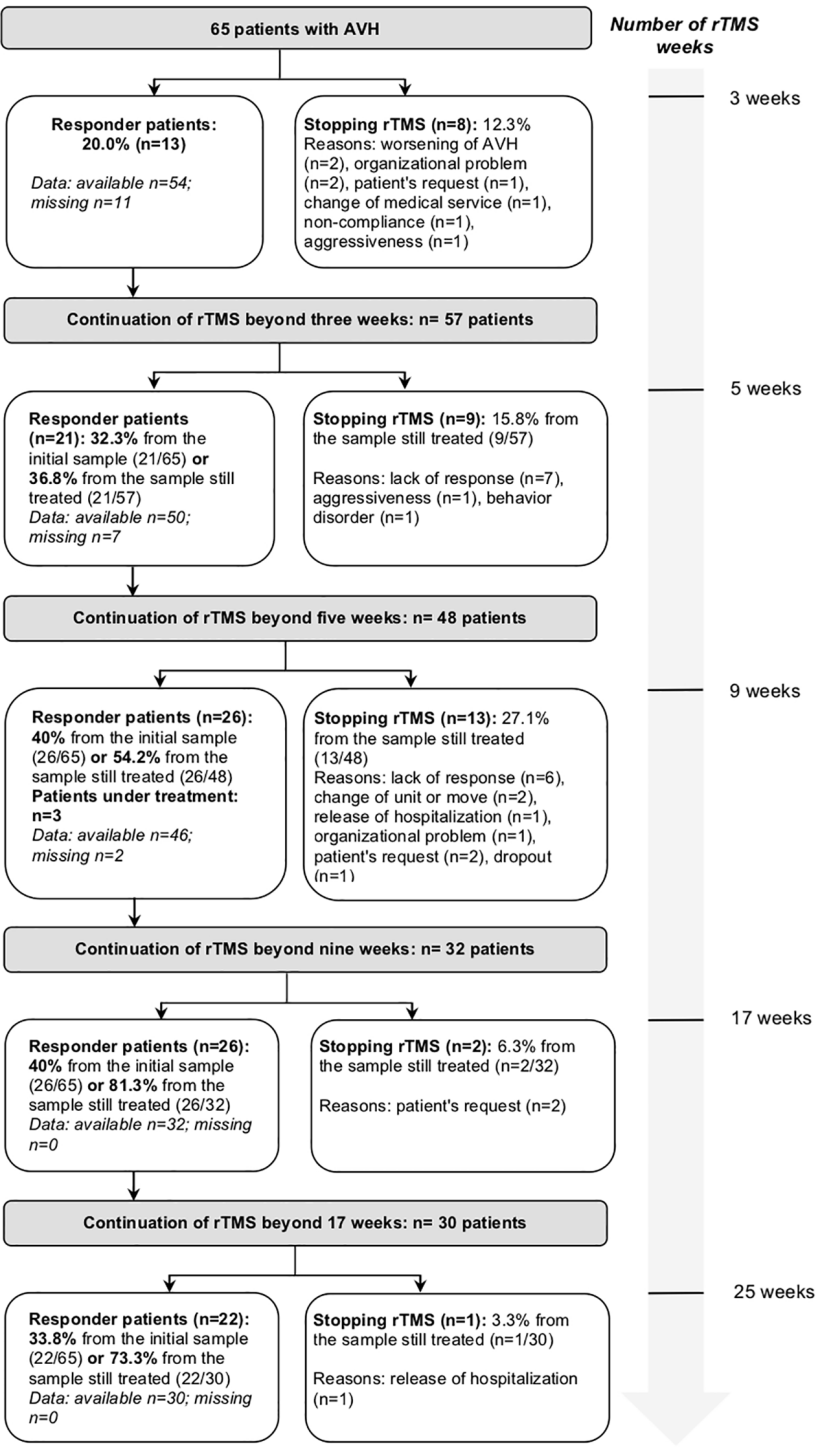
Flow chart of patients treated with repetitive transcranial magnetic stimulation over the weeks. rTMS, repetitive transcranial magnetic stimulation; AVH, auditory verbal hallucinations.

Clinical data was collected for 25 weeks. On average (SD), patients were treated by 37.2 (19.3) rTMS sessions (median=36; range= [8;84]. The course duration mean (SD) was 3.6 (2.3) months (median=4; range=[15 days; 6 months]. In **Figure 1**, the flow chart indicated the number of patients treated, the response rate and the reasons for discontinuation of treatment at the different measurement times (baseline, after three, five, nine, 17 and 25 weeks of rTMS). The maximum response rate (40%) was obtained after nine weeks of rTMS (on average (SD), after 30.6 (7.8) sessions).

Clinical scores scales (AHRS, BPRS, CGI) and the number of rTMS sessions received by patients are reported in **Table 2**. There was a significant decrease in AHRS (F[2.3;51.3]=37.1; *p*<0.001) and BPRS (F[3.0;65.9]=74.01; *p*<0.001) scores over time (from baseline up to six months). CGI-Improvement score significantly decreased over time, reflecting clinical improvement (F[2.1;39.7]=18.45; *p*<0.001).

**Table 2:** Evolution of clinical variables over the weeks of repetitive transcranial magnetic stimulation.

Variables	Before rTMS	After 3 weeks of rTMS	After 5 weeks of rTMS	After 9 weeks of rTMS (M2)	After 17 weeks of rTMS (M4)	After 25 weeks of rTMS (M6)
**Data available**	n=65	n=54	n=50	n=46	n=32	n=30
**Missing data**		n=11	n=7	n=2	n=0	n=0
**Number of rTMS sessions**	**-**	10.6 (1.9) [8; 12]	17.5 (3.4) [12; 20]	30.6 (7.8) [16; 36]	43.5 (11.5) [22; 52]	52.6 (15.4) [26; 84]
**AHRS**	29.4 (5.3) [15; 39]	23.5 (7.4) [0; 35]	22.6 (7.2) [0; 38]	18.7 (8.3) [0; 37]	11.8 (9.4) [0; 26]	12.9 (9.5) [0; 26]
**AHRS change (%)**	–	17.8% (22.9) [-13.3%; 100%]	24.2% (21.8) [-23.1%; 100%]	35.3% (29.7) [-60%; 100%]	58.8% (32.7) [10.3%; 100%]	54.4% (32.3) [10.3%; 100%]
**BPRS**	43.6 (14.3) [12; 84]	36.8 (11.6) [9; 63]	32.9 (11.7) [9; 59]	31.1 (12.6) [9; 67]	26.5 (10.7) [10; 52]	25.7 (9.9) [10; 50]
**BPRS change (%)**		13.1% (12.2) [-16.1%; 46.4%]	21.9% (18.1) [-18.2%; 72.7%]	27.7% (20.0) [-38.5; 72.7]	39.0% (17.2) [7.7; 69.7]	39.6% (14.9) [16.7; 69.7]
**CGI-Improvement**	–	2.8 (0.9) [1; 4]	2.4 (1.0) [1; 4]	2.1 (0.8) [1; 4]	1.6 (0.7) [1; 3]	1.6 (0.7) [1; 3]
*Much improved to very much improved (score <3)*		35.2% (n=19)	n=27	n=32	n=26	n=23
*Minimally improved (score=3)*		40.7% (n=22)	n=14	n=10	n=4	n=3
*No change (score= 4)*		24.1% (n=13)	n=8	n=3	n=0	n=0

Mean, standard deviation (in parentheses) and range (in brackets) are reported. AHRS, Auditory Hallucination Rating Scale; BPRS, Brief Psychiatric Rating Scale; CGI-Improvement, Clinical Global Impression-Improvement. Missing data included drop-outs and uncollected data.

Over the 25 weeks of follow-up, 31 patients stopped prematurely rTMS (47.7% of the sample) for the following reasons: lack of response (n=18, 27.7%), organizational difficulties (n=6, 9.2%), patient’s request (n=5, 7.7%) and non-compliance or lost to follow-up (n=2, 3.1%).

### 3.3 Comparison between responders and non-responders

Baseline sociodemographic and clinical characteristics of patients were compared between responders (n=26) and non-responders (n=39) after nine weeks of rTMS (**Table 1**). The treatment duration of nine weeks was chosen because it corresponded to the maximum number of responders in the sample. There was a significant effect for age: the responders were significantly younger than non-responders (p=0.002). No significant difference was found for other variables.

### 3.4 Safety and tolerability

No serious adverse events were reported during rTMS sessions.

## 4 Discussion

To our knowledge, this study is the largest to assess the effectiveness of high-frequency rTMS over the left temporo-parietal region in patients with AVH. We observed that 20-Hz-rTMS in daily practice resulted in a significant improvement of AVH. The maximum responder rate (40% of the initial sample) was reached after nine weeks of rTMS, corresponding to approximately 31 sessions in two months. The average (SD) decrease in AHRS was 35.3% (29.7). This decrease even reached 58.8% (32.7) after 17 weeks of rTMS (about 44 sessions in four months) among the 32 patients still treated at that time. After three weeks of rTMS (about 10 rTMS sessions), our responder rate (20%) was lower than that of the Dollfus et al. (17)’s RCT at Day 21 after four rTMS sessions (26.9%). However, our responder rate then increased over the sessions, reaching 40%. This clinical improvement is notable because the patients suffered from AVH resistant to antipsychotic treatment, or even to clozapine for 75% of them.

Overall, when the patients responded to treatment, the sessions were maintained but spaced out. For the patients of our sample with less severe symptoms (n=22 patients with baseline CGI severity score ≤4), rTMS sessions spaced out from the second week, with two sessions a week (instead of four). However for 14 of them (63.6%), the frequency had to be increased to four sessions per week again from the third week (n=7) or the fifth week (n=7), due to a resurgence of symptoms. This raises questions about the relevance of increasing the duration of the rTMS initial phase compared to Dollfus et al. (17)’s study. Decreasing the frequency from the first week seems indeed premature. It can be assumed that it would be more effective if the initial phase was maintained for four to nine weeks. An alternative could also be that the number of rTMS sessions per week or per day increase. In addition, the spacing of the rTMS sessions should be very progressive. So, this could allow to improve rTMS response and avoid relapses. Given schizophrenia is considered as neurodevelopmental disorder with alterations in brain circuit (22), a significant number of rTMS sessions during several weeks/months is necessary to modify these dysfunctional neuronal circuits. This long duration of care in patients with resistant schizophrenia is not specific to rTMS. For electroconvulsive therapy (ECT), the duration of treatment was at least four to six months and even in this case, a high relapse rate was reported in the weeks to months after ECT cessation (23). In order to decrease the risk of relapse, an ECT duration ranging from 6 to 12 months is now envisaged (24). However, discontinuation of neurostimulation treatment should be considered after a certain period of time. Reading our results, the question arises as to whether it is relevant to continue the sessions beyond the 17^th^ week. Indeed, the number of patients responding to rTMS decreased between the 17^th^ (n=26) and the 25^th^ sessions (n=22). The possible reasons for this decrease are: the patient’s wish to stop rTMS (n=1), spacing of rTMS sessions (n=1), lack of compliance (n=1). No specific reason has been identified to explain the poorer response of the fourth patient. It cannot be excluded that a blunting of the effect of rTMS may occur after several months.

Interestingly, the very good tolerance of the rTMS treatment allowed excellent compliance: only seven patients (10.8%) asked to stop rTMS or were non-compliant, which is a very low rate in this population of chronic and resistant patients. In addition, the therapeutic benefit felt by the patients was such that several of them did not want to stop or space out the sessions, which explains the large number of sessions administered (≥ 40 sessions in 27.7% of the sample) and the duration of the course (≥ 4 months in 43.1% of the sample). The fact that patients with schizophrenia wished to continue treatment is rare enough to be noted.

Regarding the rTMS target, it was placed without neuronavigation, unlike the first studies that used high-frequency rTMS in HAV (16, 17). Performing an MRI can indeed be complicated in these treatment-resistant patients. Although neuronavigation targeting provides precision and reliability, effectiveness of rTMS does not seem inferior in our study, which shows its feasibility and interest in routine care. However this point should be tested in a RCT.

Finally, the only factor that seemed to distinguish responders from non-responders was age, confirming Koops et al. (12)’s study which found that younger patients had better outcomes. This finding might reflect higher brain plasticity in young people (25), which facilitates the induction of long-term depression by rTMS in stimulated brain areas (12). Another hypothesis would be that the impact of age on response is mediated by cortical atrophy (26), but the relatively young age of our patients does not really support this. On the other hand, baseline clinical scores, gender, laterality and concomitant pharmacological treatment did not seem to affect clinical outcomes.

The limitations of this study are inherent to its retrospective and naturalistic design and to the lack of a control group. First, the study design (open-label, non-randomized, and uncontrolled) did not allow us to conclude on the efficacy of rTMS and to determine the optimal initial and maintenance phases duration, number of rTMS sessions and their frequency. In addition, the clinical improvement could also be explained by the placebo effect and the patient expectations. Any changes of the nature or dose of concomitant medication during the rTMS course could explained clinical improvement rather than the neuromodulatory effects of rTMS. However, as patients were generally in a state of therapeutic impasse, their pharmacological treatments remained relatively stable over the study period.

Moreover, the total number of rTMS sessions, the duration of the course and the frequency of sessions depended on the clinical condition of patients and so, were variable from one patient to another, making it difficult to draw overall conclusions. Even if it is methodologically questionable, it is part of a personalized medicine approach, which adapts to the patient’s response and wishes. In addition, as the assessments were conducted within the framework of routine clinical practice and repeated at regular intervals, a comprehensive evaluation of the patients, particularly concerning negative symptoms and cognitive deficits, could not be performed. However, these factors may have influenced the treatment response and should be considered in future studies. Regarding response criteria, the threshold of 30% reduction in the AHRS could be considered arbitrary. In addition, AHRS fails to take into account the temporal fluctuation of AVHs. In future studies, patients could self-assess their AVHs at the time they occurred, enabling to report variations in AVHs over time. For example, the Self-assessment scale of auditory verbal hallucinations (SAVH) could provide a complementary measure of the efficacy of treatments targeting AVHs (27).

Regarding the follow-up period, 25 weeks were probably insufficient to assess long-term effect of rTMS. However, this follow-up was twice as long as the studies with the longest follow-up, that were included in the recent meta-analysis assessing the effect of rTMS on auditory hallucinations in schizophrenia (14). At last, the monocentric design limited the external validity of the findings and their applicability to broader clinical populations.

For future placebo-controlled studies, it would be useful to assess the efficacy of high-frequency rTMS in HAV with an initial phase of several weeks and a maintenance period of several months and to determine the optimal duration of each of these phases. The most effective number of sessions per day and per week should also be the subject of prospective well-designed study. At last, the sample size of future studies must be calculated with a sufficient power to conduct robust subgroups analyses, such as responders versus non-responders. Conducting a multicenter study will allow for the inclusion of larger sample sizes.

## 5 Conclusion

High-frequency rTMS over the left temporo-parietal region resulted in a significant improvement of AVH, even in the absence of neuronavigation. Younger patients may have more chances of responding after receiving rTMS. These data show the clinical interest of rTMS in daily practice in patients with schizophrenia suffering from AVH. In order to obtain clinical response in a maximum number of patients knowing that they are resistant to antipsychotic treatments, or even to clozapine, it seems that the duration of the treatment must be revised upwards compared to the first studies.

## Data Availability

The raw data supporting the conclusions of this article will be made available by the authors, without undue reservation.
